# Genotype-Corrector: improved genotype calls for genetic mapping in F_2_ and RIL populations

**DOI:** 10.1038/s41598-018-28294-0

**Published:** 2018-07-04

**Authors:** Chenyong Miao, Jingping Fang, Delin Li, Pingping Liang, Xingtan Zhang, Jinliang Yang, James C. Schnable, Haibao Tang

**Affiliations:** 1 Center for Genomics and Biotechnology, Fujian Provincial Key Laboratory of Haixia Applied Plant Systems Biology, Haixia Institute of Science and Technology (HIST), Fujian Agriculture and Forestry University, Fuzhou, 350002 China; 2 Center for Plant Science Innovation, Department of Agronomy and Horticulture, University of Nebraska-Lincoln, Lincoln, NE 68588 USA; 3 Data2Bio LLC, 1111 WOI Road, Ames, IA 50011 USA

## Abstract

F_2_ and recombinant inbred lines (RILs) populations are very commonly used in plant genetic mapping studies. Although genome-wide genetic markers like single nucleotide polymorphisms (SNPs) can be readily identified by a wide array of methods, accurate genotype calling remains challenging, especially for heterozygous loci and missing data due to low sequencing coverage per individual. Therefore, we developed Genotype-Corrector, a program that corrects genotype calls and imputes missing data to improve the accuracy of genetic mapping. Genotype-Corrector can be applied in a wide variety of genetic mapping studies that are based on low coverage whole genome sequencing (WGS) or Genotyping-by-Sequencing (GBS) related techniques. Our results show that Genotype-Corrector achieves high accuracy when applied to both synthetic and real genotype data. Compared with using raw or only imputed genotype calls, the linkage groups built by corrected genotype data show much less noise and significant distortions can be corrected. Additionally, Genotype-Corrector compares favorably to the popular imputation software LinkImpute and Beagle in both F_2_ and RIL populations. Genotype-Corrector is publicly available on GitHub at https://github.com/freemao/Genotype-Corrector.

## Introduction

With the availability of high-throughput sequencing (HTS) technology, it is now straightforward to identify and score large numbers of SNP variants segregating in mapping populations. Many methods combined with HTS have been used to discover and score genome-wide SNP markers, including reduced representation sequencing^1^, genotyping-by-sequencing (GBS)^2,3^, restriction site-associated DNA sequencing (RAD-seq)^4,5^, multiplexed shotgun genotyping (MSG)^6^, and whole-genome re-sequencing^7^. These genotyping protocols offer different trade-offs between marker numbers, accuracy, and cost-effectiveness, all of which influence the statistical power to resolve recombination events and compute genetic distances when constructing genetic maps.

In practice, missing data and incorrect genotype calls can both increase the level of difficulties of constructing a genetic map and decrease the accuracy of the end product. For methods based on WGS or targeted sequencing, large genome size and sub-optimal amounts of sequencing data generated per sample (as a result of limited budgets) can produce a relatively low depth of coverage at certain loci. Such low sequencing coverage often leads to inaccurate genotype calls, especially in heterozygous regions which require deeper coverage to identify both alleles^8^. Previous studies have shown that genotyping errors could lead to erroneous map orders and an inflation of map lengths, especially when marker density increases^9^. Similarly, high rates of missing data may also have a significant impact on the constructed maps^10^.

In order to improve genotype data quality, especially data that is collected using low-coverage WGS or GBS, many genotype imputation methods have been developed such as Beagle^11^ and IMPUTE^12^. These methods are very powerful and have been widely used in human population studies^13–16^. There are also some approaches designed specifically for plant studies. For example, LinkImpute^17^ and FILLIN^18^ are optimized for low-coverage sequencing data in plants^19,20^. All of this software uses robust statistical methods to address the missing data problem in diverse populations and can also be adapted to more structured populations such as F_2_ or RIL. However, very little efforts are devoted to addressing the problem of incorrect genotype calls.

In the earlier days of application of HTS in population studies, there was a sliding-window-based method developed for high-throughput genotyping in a rice RIL population^7^. Although the method is now outdated with respect to genotype calling, the core idea of this method is applicable to the imputation of missing data and correction of wrong genotype calls for low-coverage sequencing data. However, the approach is constrained by a number of factors. First of all, the approach is not able to deal with heterozygous calls in the input data, limiting its utility to only advanced generation RIL populations, which typically require much more resources to generate. Secondly, it assumes reference genome sequences to be known for both parents, which was feasible for their particular rice population, but cannot be generalized to other plants. Thirdly, the approach assumes certain error rates for each type of genotype call prior to error correction, which can be difficult for researchers to determine beforehand. Finally, there is no publicly available software so ultimately there is a usability issue.

Here, we describe our software, Genotype-Corrector, based on the sliding window approach that addresses many of the issues described above. Genotype-Corrector offers an easy-to-use command line interface and can be applied in both F_2_ and RIL populations. We also developed a pipeline based on Genotype-Corrector and utility scripts to simplify genetic map construction. Our results showed that Genotype-Corrector can improve the accuracy of segregation datasets in F_2_ and RIL populations genotyped by mainstream genotyping methods. The constructed linkage groups using our corrected genotype data are much cleaner compared to the original linkage groups and any significant distortions were corrected as a result of running the software.

## Results

### Accuracy of Genotype-Corrector in synthetic datasets

We evaluated the accuracy of Genotype-Corrector by using a simulated F_2_ population and a *Medicago truncatula* RIL population. The simulated F_2_ population was composed of 120 individuals, with six diploid chromosomes containing 1200, 1200, 1080, 1000, 640 and 600 SNP markers respectively. The number of recombination breakpoints in each chromosome was simulated according to a Poisson distribution with λ = 1 which meets the general expectation of one cross-over per chromosome per generation. Following the initial simulation, the percentage of the three genotypes ‘A’ (homozygous locus where the allele is inherited from one parent), ‘X’ (heterozygous locus with alleles from both parents), and ‘B’ (alternative homozygous locus where the allele is inherited from the other parent) were 24.3%, 50.3%, and 25.4% respectively, consistent with the expectation of genotype ratios in a typical F_2_ population. The genotype data of the RIL population was derived from the *Medicago truncatula* genome project^3^. This RIL population included 139 individuals sequenced using GBS technology and 12,002 SNP markers were called. We masked all missing data and corrected false genotype calls based on the reported recombination breakpoints. The percentage of ‘A’, ‘X’, ‘B’ were 47.8%, 4.4%, and 47.8% respectively, consistent with the expectation of a typical RIL population advanced to the F5 or F6 generations.

Missing calls and false heterozygous calls are two types of noise that were artificially introduced into these datasets. Missing calls are the cases in which no genotype calling is made for a given SNP site in an individual, often due to lack of read coverage. False heterozygous calls are those loci where genotypes are heterozygous but are falsely called as homozygous because only one of the two alleles are present. These two issues are the most common problems encountered in constructing genetic maps using genotype calls from low coverage sequencing data. Both the missing data and heterozygous errors, ranging from 0–100% in steps of 2.5%, were introduced into the F_2_ and RIL genotype datasets. In total, we generated 1600 (40 × 40) genotype datasets in F_2_ and RIL populations for testing.

We ran Genotype-Corrector on each dataset and calculated the true positive rate (TPR), true negative rate (TNR) and accuracy (ACC), defined as:

1 $${\rm{TPR}}=\frac{{\rm{No}}{\rm{.}}\,{\rm{of}}\,{\rm{false}}\,{\rm{genotypes}}\,{\rm{corrected}}}{{\rm{No}}{\rm{.}}\,{\rm{of}}\,{\rm{false}}\,{\rm{genotype}}\,{\rm{calls}}\,{\rm{before}}\,{\rm{correction}}}$$

2 $${\rm{TNR}}=\frac{{\rm{No}}{\rm{.}}\,{\rm{of}}\,{\rm{true}}\,{\rm{genotypes}}\,{\rm{unchanged}}}{{\rm{No}}{\rm{.}}\,{\rm{of}}\,{\rm{true}}\,{\rm{genotype}}\,{\rm{calls}}\,{\rm{before}}\,{\rm{correction}}}$$

3 $${\rm{ACC}}=\frac{{\rm{No}}{\rm{.}}\,{\rm{of}}\,{\rm{true}}\,{\rm{genotypes}}\,{\rm{after}}\,{\rm{correction}}}{{\rm{Total}}\,{\rm{no}}{\rm{.}}\,{\rm{of}}\,{\rm{genotype}}\,{\rm{calls}}}$$

TPR, TNR and ACC represent sensitivity, specificity and accuracy respectively.

**Figure 1:**
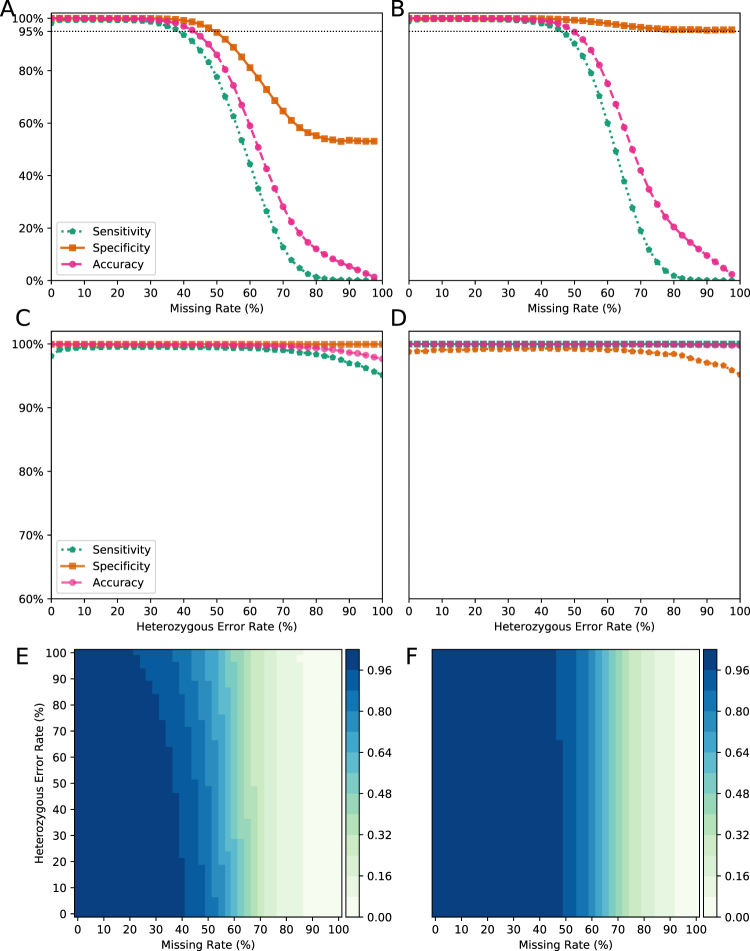
Accuracy of Genotype-Corrector on synthetic datasets. Accuracy of Genotype-Corrector with different proportions of missing data on (**A**) the simulated F_2_ population and (**B**) the RIL population. Accuracy of Genotype-Corrector with different heterozygous error rates on (**C**) the simulated F_2_ population and (**D**) the RIL population. Accuracy of Genotype-Corrector with various missing rates and heterozygous error rates on (**E**) the simulated F_2_ population and (**F**) the RIL population. Sensitivity is defined as true positive rate (TPR) and specificity is defined as true negative rate (TNR).

Our results show that the performance of Genotype-Corrector is good across a range of false genotype rates in heterozygous regions without introducing missing data both in simulated F_2_ and *Medicago truncatula* RIL populations (Fig. 1C and D). In the simulated F_2_ and *Medicago truncatula* RIL populations, the accuracy is consistently over 95% when the missing data rates stay less than 40% in F_2_ and 45% in *Medicago truncatula* RIL populations. This suggests that Genotype-Corrector can tolerate more missing data in a RIL population than an F_2_ population because of more heterozygous genotypes in the F_2_ population (Fig. 1A,B,E and F).

### Comparison with Beagle and LinkImpute

**Figure 2:**
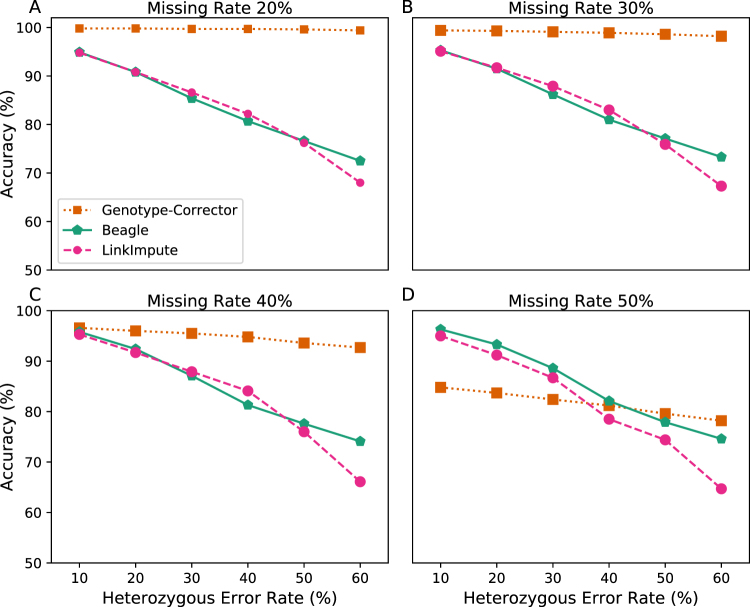
Comparison of Genotype-Corrector to LinkImpute and Beagle under different missing data rates and heterozygous error rates. Accuracy of Genotype-Corrector (brown squares), Beagle (green pentagons), and LinkImpute (pink circulars) in scenarios with different degrees of missing data (from 20% to 50%) and heterozygous error rates (from 10% to 60%).

We compared Genotype-Corrector to Beagle (v4.1)^11^ and LinkImpute (v1.1.3)^17^ that are both popular imputation tools. While both Beagle and LinkImpute are capable of imputing missing data, they do not have the ability to correct false genotype calls. To calculate the accuracy of each method under different scenarios, we performed the comparison in the simulated F_2_ population across varying factors: missing data rates in the genotype dataset from 0.2 to 0.5 with a step of 0.1 and heterozygous error rates from 0.1 to 0.6 with a step of 0.1. Then we ran Genotype-Corrector, Beagle, and LinkImpute all with their default settings. We calculated the accuracy [Equation 3] and compared the performance of these three software packages [Fig. 2].

When the missing data rates are below 40%, Genotype-Corrector consistently outperforms Beagle and LinkImpute [Fig. 2A–C]. When the missing data rate is higher than 40%, Beagle and LinkImpute start to outperform Genotype-Corrector when the heterozygous error rate is less than 40% [Fig. 2D]. Without introducing false genotypes calls, unsurprisingly, Beagle and LinkImpute have similar performances and both have high accuracy in various missing rates, reflecting their robust methods to impute missing data. Although Genotype-Corrector is sensitive to high rates of missing data, it has the greatest performance across all levels of false heterozygous error rates when missing data rates stay below 40%.

### Maize IBM RIL population

To test how the Genotype-Corrector improves the quality on real genotype datasets, we collected genome-wide SNP data of 230 RIL from the maize IBM (Intermated B73 and Mo17) population^21^. The genotypes were called with tGBS data which can provide higher sequencing coverage in heterozygous regions than the standard GBS technology^22^. Four sets of genotype calls: Homo1, Homo2, Homo3, and Homo4 were generated from the raw SNP data. The 4 genotype datasets represent varying degrees of homozygous call stringency. In Homo1, homozygous genotypes were called when at least one read was observed to support the calling. In Homo2, at least 2 reads were required to support the homozygous genotype calls, which means that when there was only one read at a certain locus, it would be treated as missing data in the Homo2 dataset. Homo3 and Homo4 were generated similarly but they required a minimum of 3 and 4 reads respectively to call homozygous genotypes. After the initial genotype calls, we filtered the markers in each dataset according to these criteria: (1) the missing rate higher than 50%, (2) the minor allele frequency (MAF) lower than 10% and (3) the heterozygous rate was more than 20%. Lowering the required numbers of reads to make a homozygous genotype call increases the total number of genotype calls, but also decreases the accuracy of genotype calls [Table 1]. To evaluate how Genotype-Corrector increases the informative content in each genotype dataset, a genotype dataset including 68 RILs which is a subset of IBM population with RNA-Seq genotyping method was used as the ground truth^23^.

**Table 1:** Comparison of four genotype datasets in IBM RIL population.

Genotype dataset	SNPs No.	Missing Rate	Accuracy
Homo1	33,131	28.5%	84.9%
Homo2	18,182	26.4%	93.5%
Homo3	13,730	26.2%	95.7%
Homo4	11,394	26.5%	96.5%

Four genotype datasets Homo1 to Homo4 were generated based on different criteria to make a homozygous genotype call. Accuracy is defined as the concordance rate [Equation 4].

The accuracy was computed by calculating the concordance rate between the tGBS genotype calls and the ground truth [Equation 4].

4 $${\rm{Accuracy}}=\frac{{\rm{No}}{\rm{.}}\,{\rm{of}}\,{\rm{concordant}}\,{\rm{sites}}\,{\rm{between}}\,{\rm{tGBS}}\,{\rm{and}}\,{\rm{ground}}\,{\rm{truth}}}{{\rm{No}}{\rm{.}}\,{\rm{of}}\,{\rm{shared}}\,{\rm{sites}}\,{\rm{between}}\,{\rm{tGBS}}\,{\rm{and}}\,{\rm{ground}}\,{\rm{truth}}}$$

**Figure 3:**
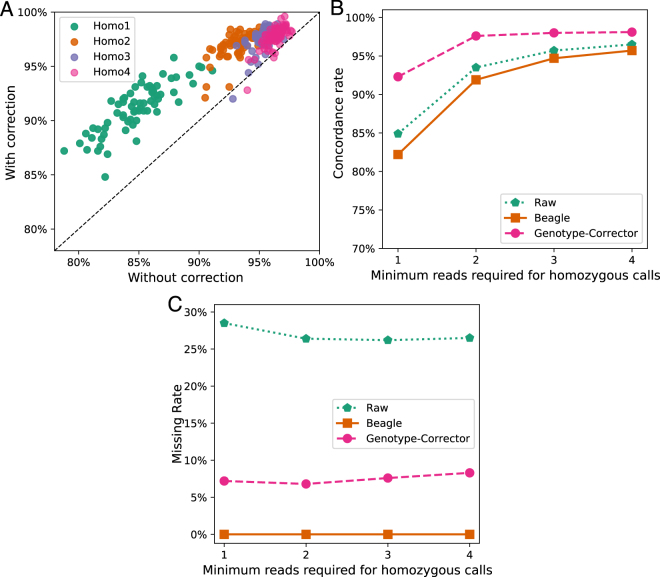
Concordance rates for corrected and uncorrected genotype datasets in maize IBM RILs. (**A**) The concordance rate between tGBS genotype calls and the ground truth before correction (raw) and after correction using Genotype-Corrector. Each dot represents a maize RIL. Green, brown, purple, and pink dots represent the genotype calls generated from raw SNP data by requiring at least 1, 2, 3, and 4 aligned reads to make a homozygous genotype call. (**B**) Comparison of concordance rates in 4 datasets after applying Genotype-Corrector (pink) and Beagle (brown). The green line indicates the concordance rates before performing correction or imputation. (**C**) Comparison of missing rates in 4 genotype datasets after applying Genotype-Corrector (pink) and Beagle (brown). The green one indicates the missing data rates in raw genotypes calls.

Each genotype dataset was corrected by Genotype-Corrector and imputed by Beagle^11^ with their default parameters. Our results show that Genotype-Corrector increased the concordance rate for each genotype dataset, especially when fewer reads were required for a homozygous call [Fig. 3A]. At the same time, Genotype-Corrector imputed most missing data while retaining high concordance rates after correction for each dataset [Fig. 3B]. In contrast, Beagle imputed all the missing data but yielded a lower concordance rate [Fig. 3B and C], suggesting that false genotype calls in the data have misled Beagle to generate erroneous results. Overall, Genotype-Corrector increased the accuracy of the genotype dataset Homo2 to an equivalent rate to that of Homo4. At the same time, the overall missing data rate in Homo2 drops from 26.2% to 6.7%.

**Figure 4:**
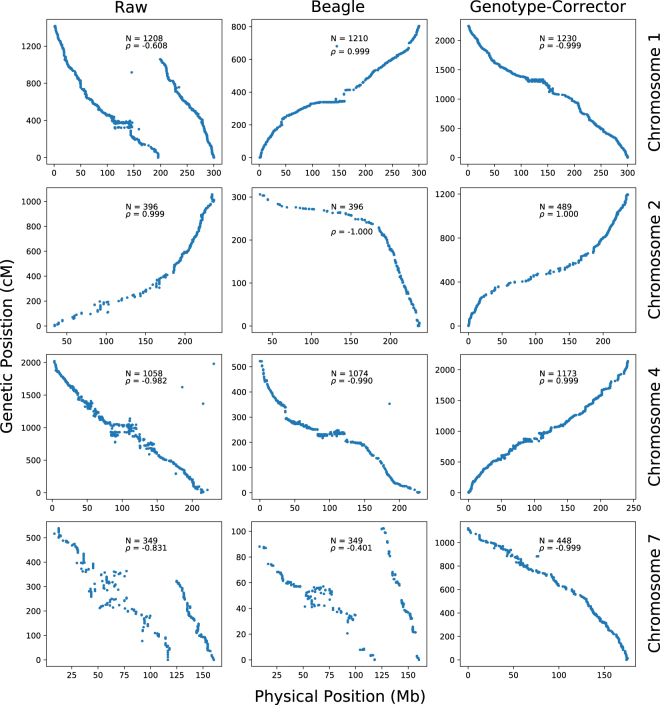
Comparison of 4 linkage groups constructed on 3 genotype datasets (raw, imputed by Beagle, corrected by Genotype-Corrector) in maize IBM RIL population. Each dot indicates the physical position of a single SNP marker on the chromosome (x-axis) versus the genetic map locations (y-axis). *N*: Number of markers involved in the linkage group; *ρ*: Spearman’s rank-order correlation measuring the concordance between the physical and genetic distances with values in the range of −1 to 1 (values closer to −1 or 1 indicate near-perfect collinearity). All linkage group results are available in the additional files^25^ and comparisons including all 10 chromosomes can be found in [Figure S2].

Using the corrected genotypes by Genotype-Corrector, a maize genetic map containing 10 linkage groups was successfully constructed using ASMap^24^ [Figure S1]. To illustrate the effect of genotype correction, we have extensively compared linkage groups constructed on 3 Homo2 genotype datasets (raw, imputed by Beagle, and corrected by Genotype-Corrector) in the IBM RIL population. The raw genotype dataset with the same parameters in ASMap yielded a map that contains a large number of errors, for example, both chromosome 1 and 7 were split into two groups. Additionally, there are synteny disruptions as well as noisy placement of markers in the reconstructed linkage groups from the raw genotypes. In comparison, the genetic map is visibly improved by imputation from Beagle, especially on chromosome 1, 6 and 10 [Figure S2]. However, while using the imputed genotypes by Beagle fixed the main problem for chromosome 1, it failed to incorporate the split groups on chromosome 7, as well as still showing a large number of noisy and distorted markers [Fig. 3]. Compared to Beagle, the linkage groups constructed based on corrected genotypes by Genotype-Corrector fixed the major issues on chromosome 7 and also showed substantially fewer noisy and misplaced markers. Overall, 4 out of 10 chromosomes included in Fig. 4 showed substantial improvements. The comparisons between the raw, imputed, and corrected set have illustrated that Beagle improved the quality of linkage groups over raw data, and that Genotype-Corrector fixed even more errors than imputation by Beagle. The complete comparisons including all 10 chromosomes can be found in [Figure S2]. All three genotype datasets and the corresponding linkage groups are available in the additional files^25^.

### Papaya F_2_ population

In order to test how Genotype-Corrector facilitates genetic map construction in real F_2_ populations which contain more heterozygous loci than RIL populations, we generated a dataset of 93 papaya (*Carica papaya*) F_2_ individuals developed from cultivars *Sunset* and *Au9*. A total of ~1.2 Gigabytes(Gb) of whole genome re-sequencing data which is equivalent to ~3x coverage of the genome was generated for each individual^26^. In this study, a new draft genome of *Sunset* was used and the contig N50 was about 395Kb [Additional files^25^]. SNPs were called using Freebayes^27^ and further filtration was performed on the raw genotype calls [See methods].

As expected, at a relatively low coverage of re-sequencing, our genotype dataset included a total of 101,962 SNP markers and the percentages of ‘A’, ‘X’, and ‘B’ were 26.1%, 35.5%, and 25.8% respectively. The missing data rate was 12.6%. Since substantially fewer heterozygous genotypes (X’s) were observed than expected, approximately 8,361 (101,962 × [(1–12.6%) × 50% − 35.5%] ≈ 8,361) heterozygous genotypes could be falsely called as homozygous. Without correction, working initially only with the raw genotype dataset, we attempted to bin markers and construct a genetic map but failed, resulting in only one amalgamated linkage group using MSTMap with different levels of *P*-value cutoffs. We also tried JoinMap (v4.1)^28,29^ but still failed to generate satisfactory results due to a large number of errors and missing data present in the raw genotype dataset.

**Figure 5:**
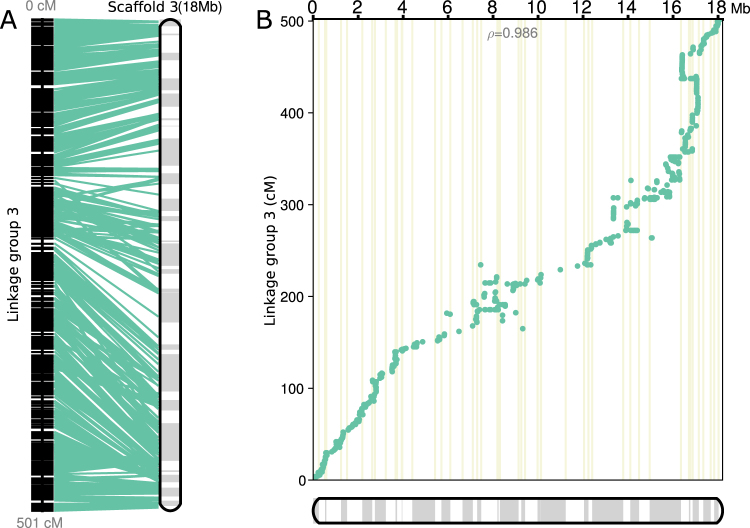
Ordering and orientations of the reconstructed papaya scaffold using ALLMAPS. (**A**) Left map contains 709 binned markers is the third papaya linkage group that we constructed. Right map is the reconstructed chromosome with physical scaffolds stitched together which include 50 contigs. Adjacent contigs within the reconstructed scaffold are shown as boxes with alternating shades. Cyan lines connect the physical positions on the reconstructed chromosome and the genetic map positions. (**B**) Scatter plot between the genetic map and reconstructed chromosome. The cyan dots represent physical positions on the chromosome (x-axis) versus the genetic map locations (y-axis). The Spearman’s *ρ* on the scatter measures the concordance between the genome assembly and the linkage map, with values in the range of −1 to 1 (values closer to −1 and 1 indicate near-perfect collinearity).

After performing Genotype-Corrector, the percentages of ‘A’, ‘X’ and ‘B’ were 23.8%, 51.2%, and 21.4% respectively, which more closely approximates the theoretical ratio of 1:2:1 than the raw data. The missing data rate also dropped substantially from 12.6% to 3.5%. Feeding the corrected genotype data to MSTMap, a papaya genetic map was successfully constructed with 15 linkage groups where 8 of them have at least 100 binned markers [Additional files^25^]. We also built a pipeline based on Genotype-Corrector to facilitate the genetic map construction. Several customized scripts involved in the pipeline can be used to preprocess genotype datasets before correction and post-process corrected genotypes [Figure S3]. Finally, we performed ALLMAPS^30^ to compute the ordering of contigs using the constructed genetic map as evidence [Fig. 5]. After anchoring of the genetic map, the contig N50 of papaya draft genome was increased from 395 Kb to 690 Kb [Additional files^25^].

## Conclusion and Discussion

For the construction of modern high-density genetic maps, researchers tend to sequence a relatively low depth of coverage for mapping individuals. This can lead to high rates of missing data and a large proportion of wrong genotype calls, especially for the heterozygous genotypes which require deeper coverage to identify both alleles. High missing rates and false genotype calls present challenges for the construction of genetic maps. To improve the quality of genotypes, researchers usually impute the missing data using imputation tools such as Beagle^11^ and LinkImpute^17^ before performing the genetic map construction. However, the issue of false genotype calls cannot be resolved by imputation only. Therefore, we developed a software package called Genotype-Corrector based on an improved sliding window approach^7^. In order to evaluate our approach in different situations, we tested the performance of Genotype-Corrector in both synthetic and real datasets. In the synthetic dataset, we found the proportion of missing data was a main factor influencing the accuracy of Genotype-Corrector. Since the correction is largely dependent on the non-missing data within a sliding window, the density of missing data has a major impact on the performance by affecting the calculation of the expected genotype probabilities (Fig. 1E and F).

By comparing Beagle, LinkImpute, and Genotype-Corrector in the simulated F_2_ and the real maize IBM RIL populations, the results indicated that Genotype-Corrector is more accurate compared to other traditional imputation tools when the missing rate is lower than 40% in both F_2_ and RIL populations [Figs 2 and 3C]. To evaluate whether the corrected genotypes improve the quality of the final genetic map construction, we compared the linkage groups constructed based on raw, imputed, and corrected genotype datasets using ASMap. Our results show that the linkage groups based on corrected genotypes contain much less noise, and have almost no apparent distortions compared to the genetic maps based on raw and imputed genotype datasets. In addition, there are more markers anchored to linkage groups, smoother genetic distance estimates and higher collinearity between physical and genetic positions were achieved with corrections by Genotype-Corrector, which indicates genotypes corrected by Genotype-Corrector can substantially improve the genetic map quality [Fig. 4, Additional files^25^].

In the more complex papaya F_2_ population, the percentage of the three genotypes in the corrected dataset more closely approximates the theoretical ratio of 1:2:1 than the uncorrected dataset, suggesting that the Genotype-Corrector can effectively correct wrong genotype calls in the heterozygous region. In order to construct the final genetic maps from the raw SNP calls, we developed an end-to-end pipeline covering pre-processing and post-processing steps [Figure S3]. This pipeline illustrates how components of Genotype-Corrector could be chained together to facilitate genetic map construction. Using this pipeline, a papaya genetic map with 15 linkage groups was successfully constructed [Additional files^25^]. Taking advantage of the constructed genetic map, the contig N50 of the papaya draft genome was increased from 395 Kb to 690 Kb using ALLMAPS, suggesting that the constructed genetic map could effectively guide the genome assembly in a *de novo* genome sequencing project [Fig. 5, Additional files^25^].

There are two main parameters in Genotype-Corrector: the error rate of the homozygous genotype calls, and the size of the sliding window. Huang *et al*.^7^ had suggested that this approach is robust to relatively high homozygous genotype error rates. For example, they estimated the error rates for two homozygous genotypes in the rice RIL population were 4.12% and 0.71%. Based on these two error profiles they achieved 99% accuracy. However, a similar accuracy result can be achieved by increasing one of the error rates to 16%.

We also found empirically that while different genotype error rates do affect the calculation of the exact probability for each genotype, these error rates often do not have a big impact on the selection of the genotype with the highest probability since the SNP error rates affect each individual probability similarly, as long as these error rates are set within a reasonable range (as dominated by sequencing errors).

The sliding window size parameter controls the granularity of the correction procedure and it largely depends on the SNP density. Although we did not control the imbalance of SNP distributions, Genotype-Corrector works well in all tested datasets with the default window size (n = 15). We also tested window sizes from 11 to 17 on maize Homo2 datasets and yielded nearly identical results [Figure S4]. In the previous study, researchers arrived at a similar conclusion by testing different window sizes from 15 to 35 as long as the SNPs density is over 25 per Mb in rice RIL population^7^. It is now routine to identify over hundreds of thousands of SNPs at reasonable costs, which also significantly weakens the influence of uneven SNP distributions.

In summary, our results suggest that Genotype-Corrector is widely applicable in whole-genome genetic mapping studies in F_2_ or RIL mapping populations. Outputs from Genotype-Corrector are compatible with popular genetic mapping software, such as MSTMap^31^, ASMap^24^, R/QTL^32^, and JoinMap^28^. Users can also generate an intermediate file where each corrected genotype is highlighted with an asterisk for debugging purposes or if users wish to further validate the automated corrected genotype calls. Although F_2_ and RIL populations are currently supported due to the availability of our test datasets, it would be straightforward to expand this approach to include more population types such as F3 or BC1F1 in the future.

## Methods

### Genotype-Corrector implementation

Prior to the genotype correction, the sequence data of hundreds of F_2_ individuals or RILs are mapped to a reference genome and SNPs are identified. The genotypes are coded as ‘A’, ‘X’, and ‘B’. In order to solve the phasing problem, when none of the two founders of the population is the reference line, one of the founders’ genotypes in each SNP site should be provided to distinguish the origin of alleles in each individual.

For F_2_ or RIL populations with no errors or contamination, genomic regions that are homozygous should contain mostly homozygous calls from one parental haplotype with some missing data. However, heterozygous loci may be misidentified as homozygous due to an insufficient number of reads to support both alleles at low sequencing coverage. When the sequencing coverage is low, it is common that two types of homozygous genotypes are distributed among a heterozygous region in a random fashion^3^. To correct false genotype calls and impute missing data resulting from low sequencing or alignment errors, a sliding window covers consecutive SNPs with a step size of one marker and the genotype with the highest expected probability was called in each window [Equations 5–7].

When we consider possible errors for each genotype call, in each window, the probability of finding *k* times of genotype ‘B’ for each genotype follows a binomial distribution. The probability of observing *k* times of genotype ‘B’ in a sliding window of size *n* can be calculated separately as the following:

5 $${P}_{A}(k)={(}_{k}^{n})\times {E}_{A}^{k}\times {(1-{E}_{A})}^{n-k}$$

6 $${P}_{B}(k)={(}_{k}^{n})\times {(1-{E}_{B})}^{k}\times {E}_{B}^{n-k}$$

7 $${P}_{X}(k)={(}_{k}^{n})\times {(\frac{1+{E}_{X}}{2})}^{k}\times {(\frac{1-{E}_{X}}{2})}^{n-k}$$

where *E*_*A*_, *E*_*B*_, and *E*_*X*_ are SNP error rates of the three genotypes. By incorporating the estimated proportions of three genotypes, the genotype with the maximum probability within the window is selected:

8 $${P}_{{\rm{\max }}}(k)=\,{\rm{\max }}\,\{{P}_{A}(k)\times {\lambda }_{A},{P}_{B}(k)\times {\lambda }_{B},{P}_{X}(k)\times {\lambda }_{X}\}$$

where the three priors – *λ*_*A*_, *λ*_*B*_, and *λ*_*X*_ are the expected Mendelian segregation ratios in the population, which can be deduced based on the population type. The expected genotype ratios within F_2_ population is 1(‘A’): 2(‘X’): 1(‘B’), with each advanced RIL reducing the expected number of heterozygotes (number of ‘X’s) by half. The process described above is repeated with several iterations until the corrected genotypes converge.

**Figure 6:**
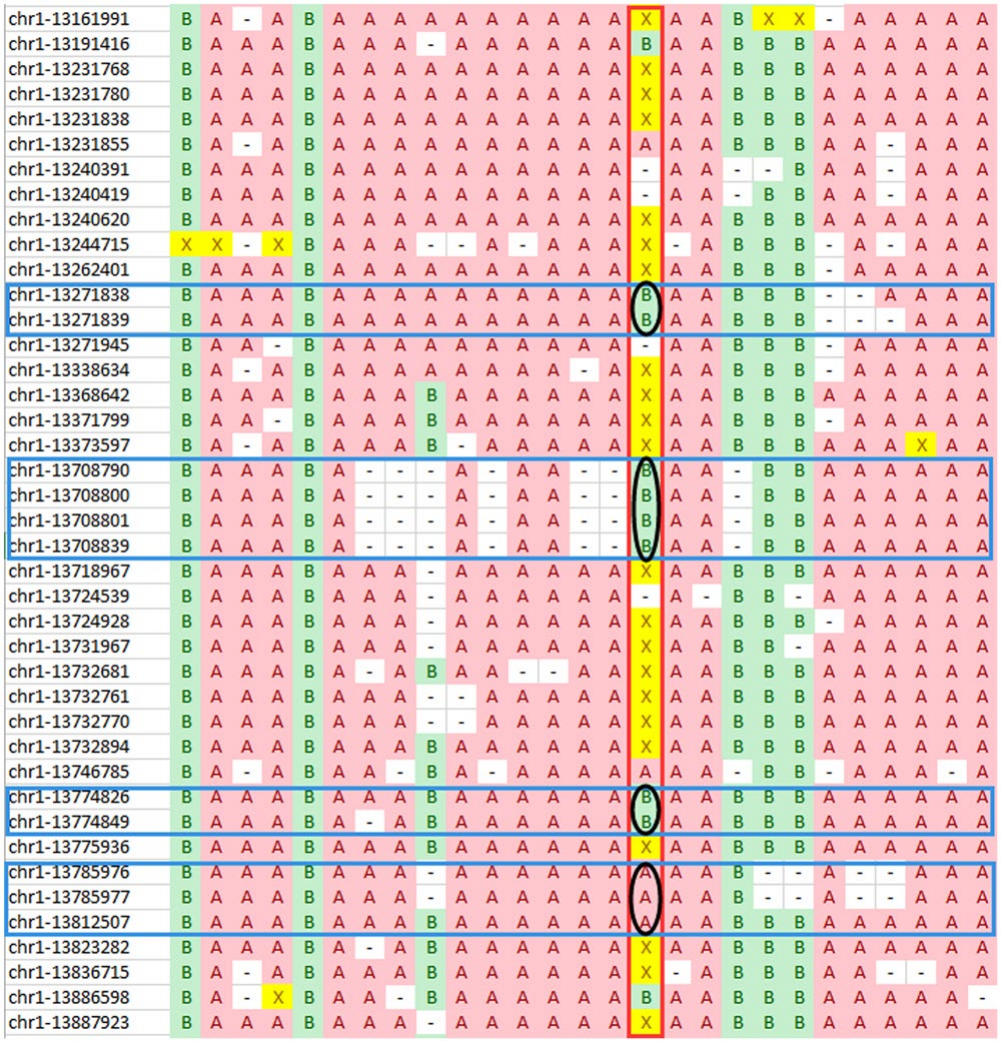
An example region illustrating the consecutive false homozygous calls within a heterozygous region. Genotype calls for markers within an interval on chromosome 1 of *Medicago truncatula* for 139 RILs extracted from the dataset in^3^. The vertical dark red frame marks a heterozygous region in an individual. Black circles and blue horizontal frames indicate that heterozygous genotypes (X’s) were incorrectly called as a stretch of identical homozygous genotypes (A’s or B’s) in a short genomic region.

When multiple segregating SNPs appear within a short genomic interval, multiple consecutive heterozygous loci (‘X’s) can be miscalled as homozygotes of the same haplotype (‘A’s or ‘B’s) [Fig. 6]. This phenomenon often occurs within a short genomic region covered by only a few reads. In practice, this apparent bias of consecutive homozygous genotypes in a heterozygous region may lead to mis-corrections when using the sliding window method. To eliminate such bias, markers that have the same consecutive homozygous genotypes within a single read length (a configurable parameter) are binned into one representative marker. This step is implemented in an independent Python script distributed with the Genotype-Corrector software package.

### Genetic map construction in maize IBM population

The Homo2 genotypes in maize IBM population was corrected by Genotype-Corrector and imputed by Beagle (v4.1) with default parameters. The raw, imputed, and corrected genotypes were converted to the corresponding ‘cross’ object which is a particular data format for R/qtl and ASMap packages. The linkage maps were constructed by using the function ‘mstmap.cross’ in ASMap with parameters: ‘p.value = 1e-13’, ‘trace = TRUE’, ‘mvest.bc = TRUE’, ‘detectBadData = TRUE’ on all three genotype datasets. Since small linkage groups without enough markers are difficult to compare between methods, only linkage groups with over 100 markers were included for comparison. Details of input data, linkage group results and scripts are available at Figshare^25^.

### Genetic map construction in Papaya F_2_ population

The papaya *Sunset* genome was sequenced using PacBio sequencing and assembled on the SMRT Portal using HGAP.3. The assembled genome size is around 341 Mb [Additional files^25^]. 93 papaya F_2_ samples were sequenced on Illumina HiSeq. 2500 with the read length 2 × 150 bp. The reads from all available samples were aligned to the draft genome using ‘bwa mem’ (v0.7.12) with default parameters^33^. Then the alignments were further processed by sorting, selecting the unique alignments, and removing the PCR duplicates. The genotype calling was performed by feeding the preprocessed alignments to Freebayes with parameters: ‘–min-alternate-count = 2’, ‘–min-alternate-fraction = 0.1’. In order to control the genotypes quality, we removed SNPs with a quality score below 30 and a missing rate over 40%. We also discarded loci with segregation distortions, where the ratio of ‘A’:‘B’ did not follow the 1:1 (*χ*^2^-test, *P*-value < 0.01). We chose 1:1 to test the ‘A’:‘B’ ratio instead of using 1:2:1 to test the ratio of ‘A’:‘X’:‘B’ because a substantial proportion of heterozygous genotypes were called as homozygous incorrectly due to a frequent failure to recover both alleles in the heterozygous regions.

Prior to the correction, the filtered genotypes were further processed by compressing the identical stretches of homozygous loci (‘A’s or ‘B’s) within the distance of the read length (150 bp) in heterozygous regions to avoid over-counting stretches of false calls. This step was implemented by the script ‘preprocess_markers.py’ distributed with Genotype-Corrector package. Then the preprocessed genotypes were corrected using Genotype-Corrector with default parameters. To obtain the genotype dataset of a size which was manageable by other software, corrected genotypes were binned, resulting in a decrease from 46,974 markers into 6,615 bins using our script ‘bin_corrected_markers.py’. The linkage groups were constructed using MSTMap on binned genotypes with parameters: ‘cut_off_p_value = 1e-16’, ‘distance_function = kosambi’, ‘objective_function = COUNT’. To use ALLMAPS anchoring contigs to scaffolds using constructed genetic map, the format of the genetic map generated from MSTMap was converted to BED format which is the standard input for ALLMAPS. This process was implemented by using the function ‘merge’ in ALLMAPS package. The converted BED file and the original contigs FASTA file were then used as the input for the ‘path’ command in ALLMAPS [Additional files^25^].

### Data availability

All the key intermediate, final results and running codes are available on Figshare (10.6084/m9.figshare.6179231.v1).

## Electronic supplementary material

Supplementary Figures
