# Supplementary material for: Genotype-Corrector: improved genotype calls for genetic mapping in F2 and RIL populations
Source: Sci Rep. 2018 Jul 4;8:10088. doi: 10.1038/s41598-018-28294-0 (PMC6031647; doi:10.1038/s41598-018-28294-0)
Supplement: Supplementary file 1 — Supplementary Figures [file 41598_2018_28294_MOESM1_ESM.pdf]

Genotype-Corrector: improved genotype calls for genetic mapping in F_2 and RIL populations

Chenyong Miao[1,2], Jingping Fang[1], Delin Li[3], Pingping Liang[1], Xingtan Zhang[1], Jinliang Yang[2], James C. Schnable[2], and Haibao Tang[1,*]

[1]Center for Genomics and Biotechnology, Fujian Provincial Key Laboratory of Haixia Applied Plant Systems Biology, Haixia Institute of Science and Technology (HIST), Fujian Agriculture and Forestry University, Fuzhou 350002, China.
[2]Center for Plant Science Innovation, Department of Agronomy and Horticulture, University of Nebraska-Lincoln, Lincoln, NE 68588, USA.
[3]Data2Bio LLC, 1111 WOI Road, Ames, IA 50011, USA.
[*]tanghaibao@gmail.com

Maize genetic map after correction

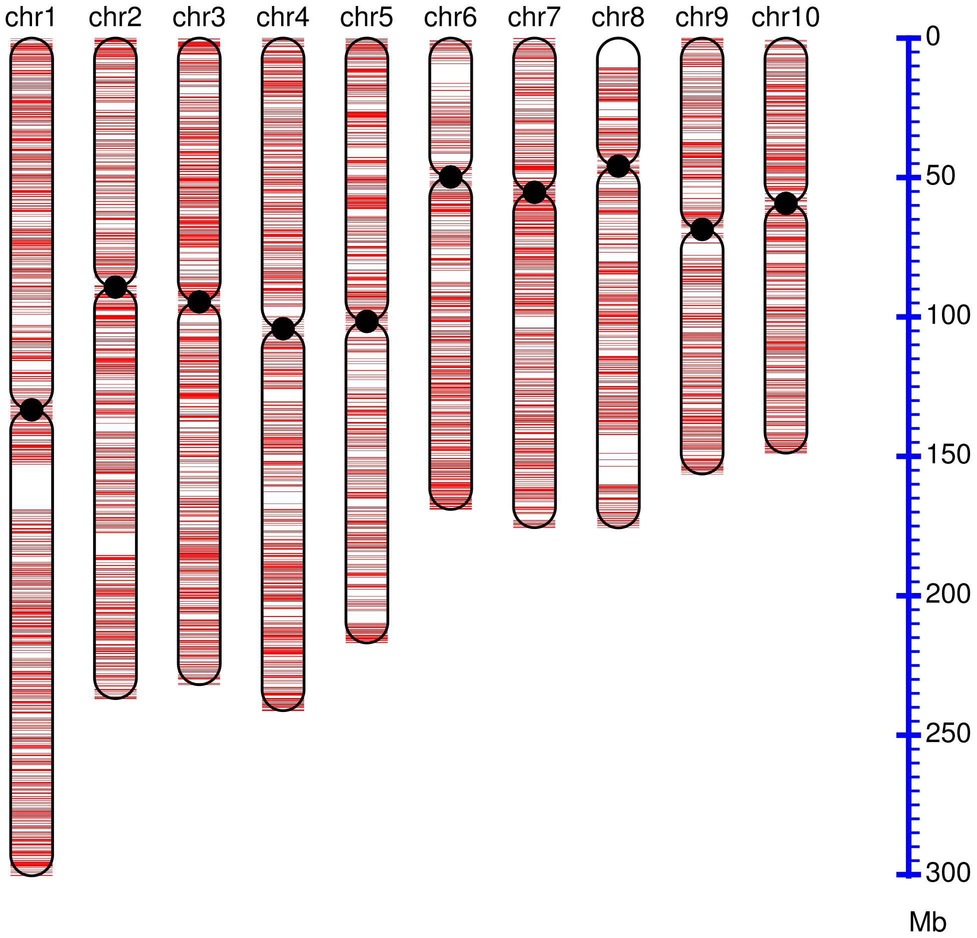

Figure S1. Maize genetic map after applying Genotype-Corrector. genotype dataset requiring at least 2 reads (Homo2) for the homozygous calls was used to construct genetic map. This dataset contains 14,638 SNPs and 230 individuals and was corrected using Genotype-Corrector with default parameters. After correction, the corrected data was fed to ASMap and 10 chromosomes as expected were generated successfully.

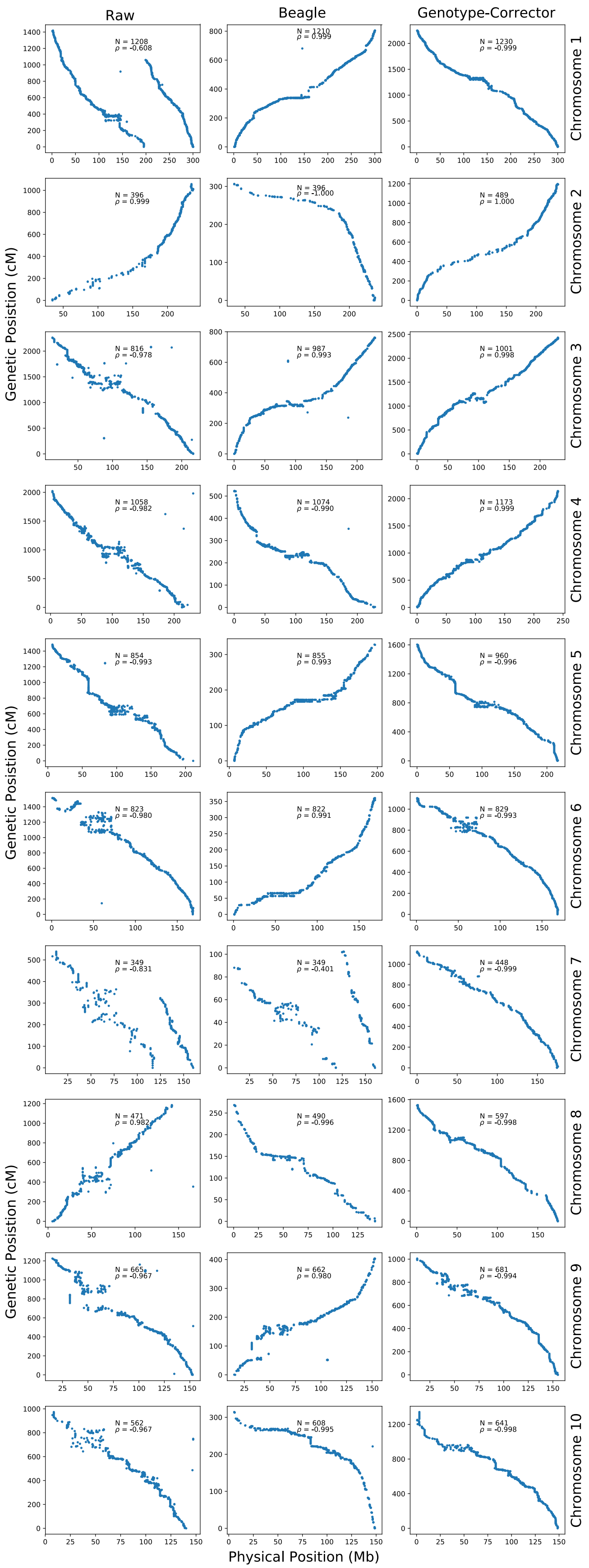

Figure S2. Comparison of 10 linkage groups constructed on 3 genotype datasets (raw, imputed by Beagle, corrected by Genotype-Corrector) in maize IBM RIL population. Each dot indicates the physical position of a single SNP marker on the chromosome (x-axis) versus the genetic map locations (y-axis). N: Number of markers involved in the linkage group; ρ: Spearman's rank-order correlation measuring the concordance between the physical and genetic distances with values in the range of -1 to 1 (values closer to -1 or 1 indicate near-perfect collinearity).

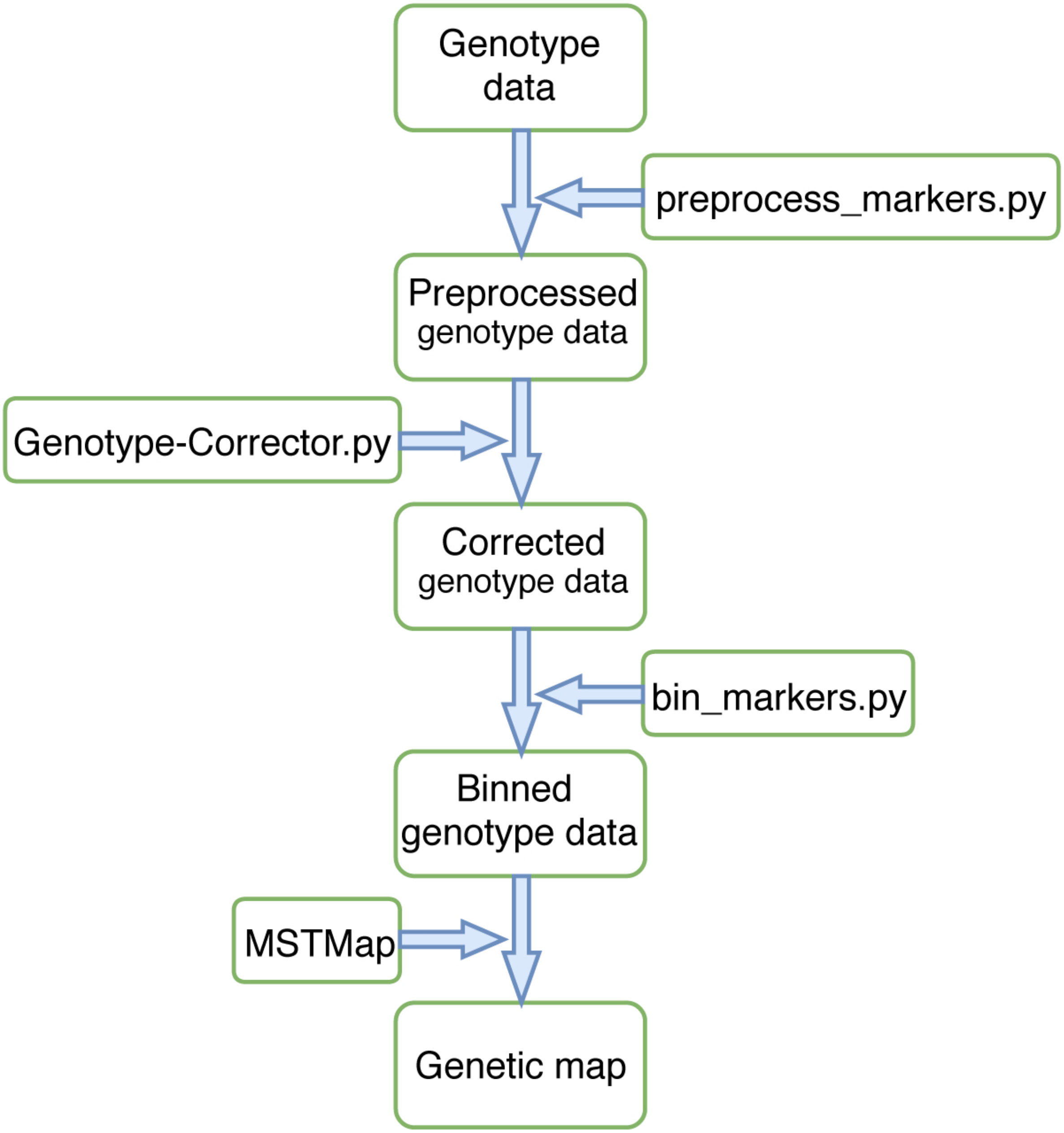

Figure S3. The work flow for constructing the papaya genetic map using Genotype-Corrector. First, the genotype dataset was preprocessed using ‘preprocess_markers.py’. This script removed identical stretches of homozygous loci (‘A’s or ‘B’s) within very short (read-length) distance in heterozygous regions to avoid over-counting stretches of false calls. Second, the genotype data was corrected using ‘Genotype-Corrector.py’. For optimal computational efficiency, corrected genotypes were combined using ‘bin_markers.py’. Finally, the corrected and binned genotype data was consumed by MSTMap and 15 linkage groups were constructed.

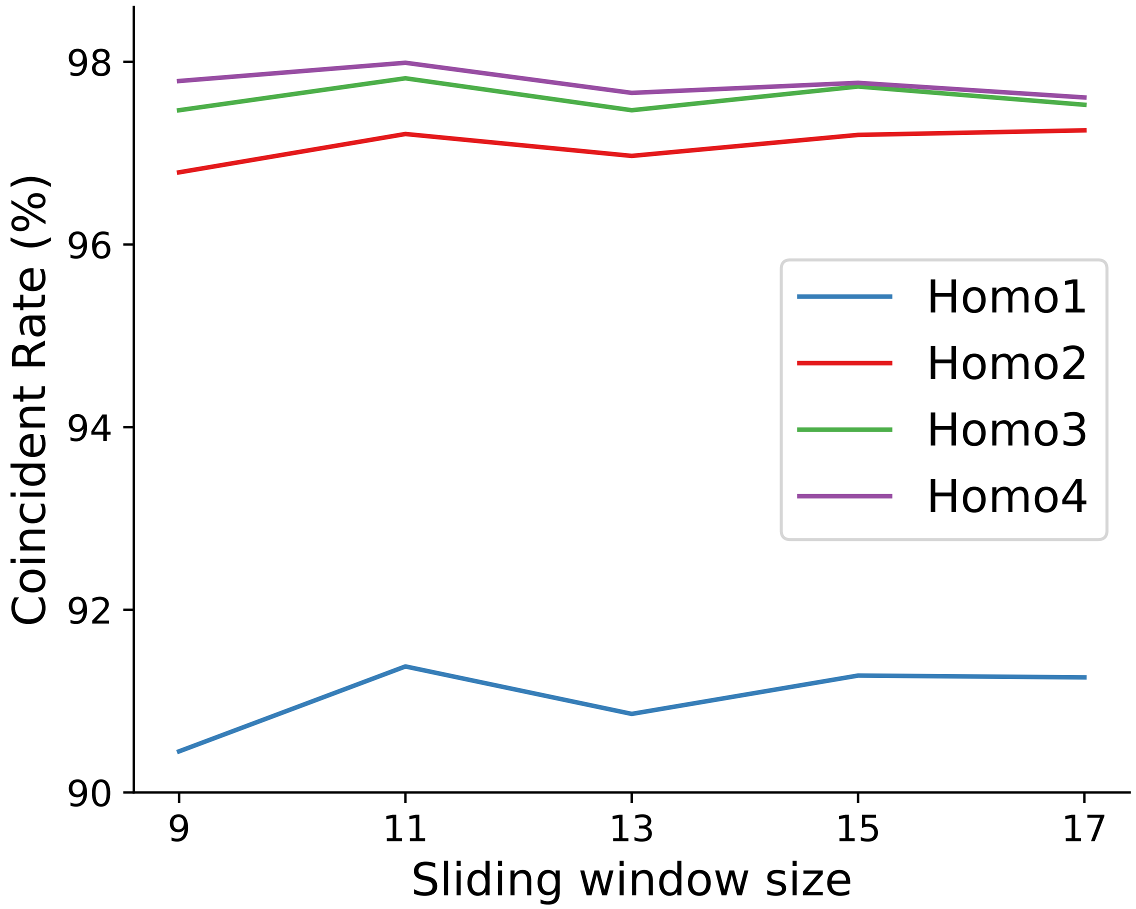

Figure S4. The effect of sliding window size on accuracy in maize RIL population. Four genotype datasets from the maize RIL population were corrected by Genotype-Corrector using different values of the window size parameter. The accuracy was measured by the concordance rate.
